# The Emerging Role of CD244 Signaling in Immune Cells of the Tumor Microenvironment

**DOI:** 10.3389/fimmu.2018.02809

**Published:** 2018-11-28

**Authors:** Laura Agresta, Kasper H. N. Hoebe, Edith M. Janssen

**Affiliations:** ^1^Cincinnati Children's Hospital Medical Center, Cancer and Blood Diseases Institute, Cincinnati, OH, United States; ^2^Division of Immunobiology, Cincinnati Children's Hospital Research Foundation, Cincinnati, OH, United States; ^3^Department of Pediatrics, University of Cincinnati College of Medicine, Cincinnati, OH, United States

**Keywords:** CD244, tumor immune escape, immune exhaustion (IE), signaling lymphocyte activation molecule, MDSC, CD8 cytotoxic T lymphocytes +, NK cells and cancer, tumor microenviroment

## Abstract

In cancer, immune exhaustion contributes to the immunosuppressive tumor microenvironment. Exhausted immune cells demonstrate poor effector function and sustained expression of certain immunomodulatory receptors, which can be therapeutically targeted. CD244 is a Signaling Lymphocyte Activation Molecule (SLAM) family immunoregulatory receptor found on many immune cell types—including NK cells, a subset of T cells, DCs, and MDSCs—that represents a potential therapeutic target. Here, we discuss the role of CD244 in tumor-mediated immune cell regulation.

## Introduction

In cancer, immune exhaustion contributes to the immunosuppressive tumor microenvironment. Immune exhaustion is defined by poor effector function with decreased pro-inflammatory cytokine production and diminished cytolytic activity caused by soluble and membrane-associated stimuli derived from tumor and immune cells. In the last decade, research into mechanisms underlying immune exhaustion, particularly in T cells, has revealed a variety of immunomodulatory receptors whose sustained expression is associated with chronic infection and cancer. Examples include Programmed cell Death protein-1 (PD-1) and its ligands (PD-L1 and PD-L2), Cytotoxic T-lymphocyte- Associated protein-4 (CTLA-4), Lymphocyte-activation Gene-3 (LAG-3), T-cell Immunoglobulin and Mucin domain-3 (TIM-3), and CD160. Recently, therapeutic checkpoint inhibitors that target PD-1, PD-L1, and CTLA-4 have been used successfully in a variety of cancers to ameliorate immune exhaustion and improve patient outcomes. However, treatment responses remain suboptimal for many patients and further targets for immunotherapy are needed.

CD244 is an immunoregulatory transmembrane receptor in the Signaling Lymphocyte Activation Molecule (SLAM) family that offers a potential target for immunotherapy. CD244 expression has been demonstrated on natural killer (NK) cells, γδ T cells, basophils, monocytes, a subset of CD8^+^ αβ T cells, dendritic cells (DC), and myeloid-derived suppressor cells (MDSC) (1–7). After early studies established that CD244 expression on these cell types is altered under specific pathologic conditions, more recent research has linked CD244 inhibitory signaling to the maintenance of an exhausted phenotype in NK cells and T cells in chronic infection and cancer (8–11). However, knowledge of CD244 signaling pathways still derives largely from NK cell studies, although expression of CD244 adaptor molecules differs between cell types and under various conditions (see Specific adaptor molecules) (12). Thus, further investigations are needed to elucidate CD244 signaling mechanisms in the broad repertoire of immune cells on which the receptor is found. In addition, more studies are needed to delineate the specific functions of CD244 signaling in tumor-associated immunosuppression. Here, we discuss the role of the CD244 receptor in tumor-mediated immune cell regulation.

## CD244 receptor

CD244 (2B4) was first identified on mouse NK cells and a subset of T cells that mediated non-MHC-restricted cytotoxicity.(1) Subsequently, the structures of mouse and human CD244 were elucidated, the receptor's ligand was identified as CD48, and initial observations regarding CD244 functionality were reported. Because CD244 is expressed on all NK cells (13), its signaling mechanisms have been best elucidated in these lymphocytes, although recent work suggests parallel mechanisms in other cell types (5, 6, 11, 14, 15). The following sections review the structure of CD244 and its signaling mechanisms.

### CD244 structure

CD244 is an Ig Superfamily Signaling Lymphocyte Activation Molecule (SLAM) family receptor. Like all SLAM family receptors, it is a transmembrane receptor comprised of an extracellular segment with two immunoglobulin (Ig)-like domains, a transmembrane region, and a cytoplasmic domain containing tyrosine-based motifs. Unlike other SLAM family receptors, it does not act as a self-ligand; instead, it binds CD48, a transmembrane receptor ubiquitously expressed on hematopoietic cells (16–18). Its cytoplasmic domain includes four Immunoreceptor Tyrosine-based Switch Motifs (ITSMs) that interact with a variety of specific adaptor molecules and are capable of propagating both inhibitory and activating signals (19, 20).

In mice, two isoforms of CD244 are expressed via alternative splicing: a long isoform with four ITSMs and a short isoform with only one ITSM (13, 21). In humans, two isoforms of CD244 are also expressed via differential splicing of hnRNA, but both human isoforms have identical intracellular domains with four ITSMs (22). Structurally, the human isoforms differ extracellularly by the presence or absence of five amino acids between the immunoglobulin V and C2 domains. Functionally, the shorter human isoform has increased affinity for CD48, and its engagement results in increased calcium flux and increased NK-cell mediated cytotoxicity *in vitro* (23).

### Specific adaptor molecules

**Figure 1:**
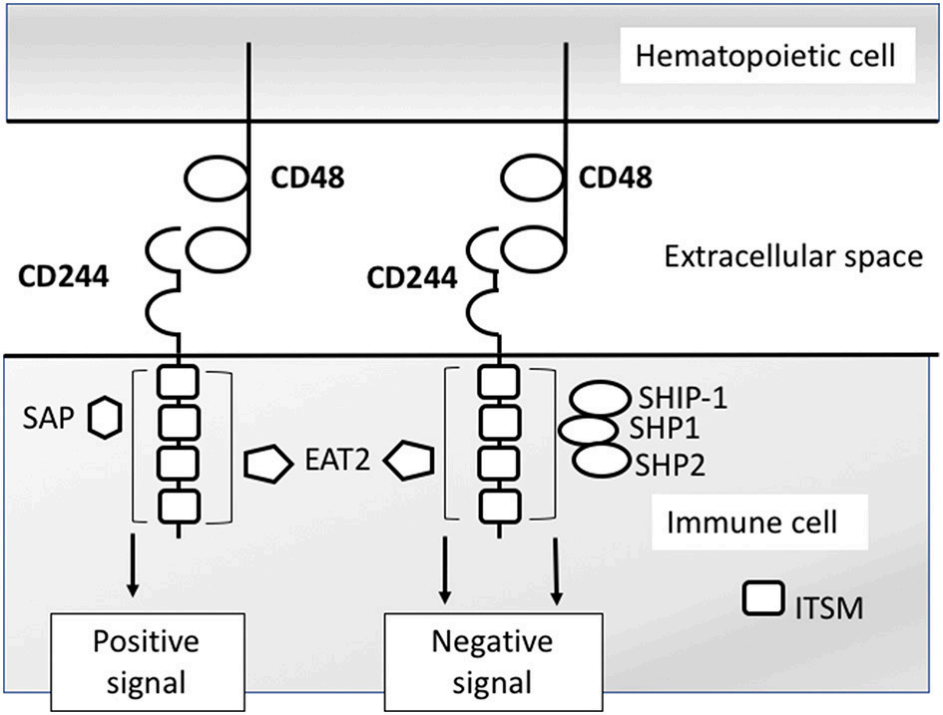
CD244 signaling model based on NK cell studies. CD244 binds CD48 with high affinity. Intracellular signaling is propagated via interactions with any of several SH2 domain- containing signaling molecules. Interactions with SAP (SH2D1A) propagate activating signals in NK cells. Interactions with SH2 phosphatases SHP1,SHP2, SHIP-1 propagate inhibitory signals in NK cells. Interactions with EAT2 (SH2D1B) have been shown to propagate both activating and inhibitors signals in separate studies.

The ITSMs of human and murine CD244 bind Src homology 2 (SH2) domain-containing proteins, including SLAM-associated protein (SAP), associated with activating signaling (24), and Ewing sarcoma-activated transcript 2 (EAT2) (25), associated with activating and inhibitory signaling (26, 27), and phosphatases SHP1 (28), SHP2 (29), and SHIP-1 (30), associated with inhibitory signaling (Figure 1). In mice only, the EAT-2-related transducer (ERT) also binds CD244 ITSMs (25). In human NK cells, the c-Src kinase (Csk) binds the second and third ITSMs (31). CD244 signaling studies in other immune cell types, which express different levels of these adaptor molecules, are lacking. Based on NK cell studies, it is thought that adaptor molecule expression levels, availability, and competitive binding determine whether CD244 propagates an activating or inhibitory signal (8, 24–27, 31–38).

CD244 can mediate activating signals in NK cells in the presence of adequate concentrations of functional SAP (24) (Figure 1). In the absence of functional SAP, CD244 is unable to initiate activating signals in mouse and human NK cells (32, 33). When SAP is unavailable for recruitment, CD244 instead recruits phosphatases (e.g., : SHP-1, SHP-2), which leads to the propagatation of inhibitory signals (8, 34–37). In subsequent investigations, specific adaptor molecule EAT-2 was also found to produce inhibitory signaling upon binding with CD244 in C57BL/6 mouse NK cells, reflected by decreased production of IFN-γ and reduced killing of targets (27). However, a later set of experiments demonstrated that C57BL/6 mouse EAT-2A^−/−^ and EAT-2A^−^/B^−^ NK cells lose CD244-specific cytotoxicity and IFNγ production compared with WT NK cells, providing evidence for an activating role (26). Of note, the initial study demonstrating inhibitory function of EAT-2 in NK cells did not demonstrate the effect of EAT-2 deficiency on CD244-CD48 mediated signaling specifically, whereas the later study demonstrated a CD244-CD48 specific effect. Comparing the function of SAP and EAT-2 in CD244 signaling, SAP is able to bind both non-phosphorylated and phosphorylated ITSMs, while EAT-2 only binds phosphorylated SLAM family ITSMs (25), which may limit the contribution of EAT-2 to the determination of activating versus inhibitory CD244 signaling. For example, in the presence of SAP, the association of inhibitory adaptor molecule SHP-2 is decreased, while EAT-2 partially inhibits the binding of SHP-2, but to a lesser degree than SAP (25, 38). Likewise, in humans, the association of CD244 with SHP-2 and SAP in transfected NK cells is mutually exclusive (38).

A mechanistic model demonstrating inhibitory signaling by CD244 in human NK cells showed that while the first, second, and fourth ITSMs of CD244 activate NK-mediated cytotoxicity by binding SAP, the third ITSM was able to bind phosphatases SHP-1, SHP-2, SHIP, and Csk, and inhibit NK cytotoxicity (31). However, only one molecule associates with the ITSM at a time, and the presence of SAP prevented binding of these phosphatases. This competitive interaction makes SAP essential to the regulation of activating versus inhibitory signaling from CD244 in human NK cells.

### CD244 expression levels and signal outcome

**Figure 2:**
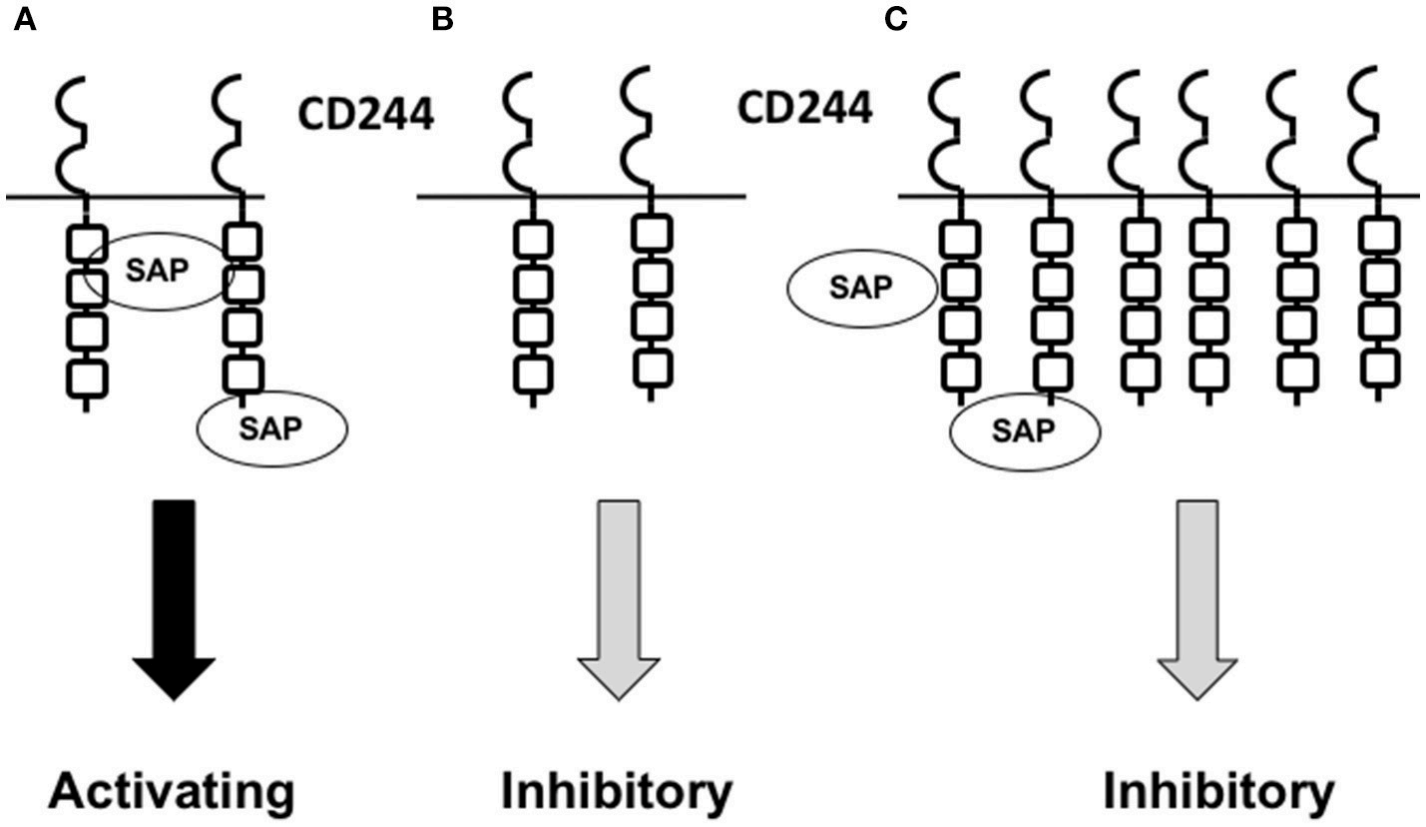
Model showing how the relative concentrations of CD244 and SAP may contribute to the determination of activating versus inhibitory CD244 signaling. **(A)** Under normal physiologic conditions, NK cells and CD8+ T cells express CD244 at low concentrations. Provided a normal intracellular concentration of SAP is present,activating signals are propagated upon CD244-CD48 interaction. **(B)** When SAP concentrations are low,absent,or dysfunctional (unable to bind), such as in X-linked proliferative disease,CD244 propagates an inhibitory signal upon CD244-CD48 interaction. **(C)** In the setting of cancer or chronic viral infection,NK cells and CD8+ T cells express high concentrations of CD244,and normal concentrations of SAP become insufficient to saturate CD244 binding sites upon CD244-CD48 interaction; an inhibitory signal is propagated.

CD244 expression is altered on different cell types under various physiologic and pathologic conditions (discussed in later sections). Alterations in the level of CD244 expression and the degree of CD244-CD48 ligation appear to contribute to determination of activating versus inhibitory signaling. CD244 has been shown *in vitro* to produce an activating function in murine NK cells when expressed at low surface levels, and an inhibitory function when expressed at high levels (39), although the pathway leading to increases in CD244 expression has not been determined. The inhibitory function can be overcome when fewer CD244 molecules are engaged or when SAP is over-expressed in transfectants expressing high surface concentrations of CD244 (39). This suggests that the CD244-to-SAP ratio is crucial in determining whether CD244 binding propagates an activating or inhibitory signal (Figure 2). The role that relative concentrations of the other CD244-associated adaptor molecules may play in determining signal type has not yet been elucidated.

## CD244 in NK cells

### Early studies of CD244 as an activating receptor in NK cells *in vitro*

NK cells are critical for surveillance and eradication of cancer cells. CD244 is expressed on all NK cells (4, 20), where its signaling mechanisms were initially characterized. Early experiments showed that treatment of mouse NK cells with anti-2B4 monoclonal antibody (mAb) led to increased IFNγ production and augmented non-MHC-restricted killing of tumor cells *in vitro* (1). In addition, CD244-CD48 homotypic interactions were shown to be essential for optimal human NK cell proliferation in response to IL-2, as well as contributing to murine NK cell proliferation, lytic potential, and cytokine secretion (40, 41). Furthermore, cross-linking of CD244 on human NK cells using anti-CD244 mAb induced NK cell-mediated lysis of target cells (2). However, the same study also found that treatment of cultured human NK cells with anti-CD244 had an antagonistic effect on IL-2-stimulated proliferation, suggesting that CD244 could mediate activating or inhibitory signaling.

### CD244 as an inhibitory receptor in NK cells *in vitro* and *in vivo*

Soon after identification of this dual functionality, it was determined that CD48+ target cells inhibit murine NK cell effector function, and blocking the CD244-CD48 interaction with CD244 or CD48 mAb relieves this inhibition, causing enhanced target cell lysis (36). In addition, CD244 preferentially accumulates at the interface between NK and target cells during non-lytic events (36). These results introduced an inhibitory role for CD244 in mouse NK cells. Findings by Lee et al. further showed that CD244 ligation inhibits NK cell-mediated lysis of CD48+ tumor cells and NK cell production of IFNγ *in vitro* (37). Correspondingly, *in vivo* administration of anti-CD244 mAb significantly decreased the number of B16F10 syngeneic melanoma lung nodules in wildtype (WT) mice following intravenous injection (42). However, female CD244^−/−^ mice showed poor rejection of both CD48(+) and CD48(–) B16 melanoma cells, suggesting a gender-based difference in these genetically-modified mice. Further studies are needed to determine whether this difference exists in other genetic backgrounds. In both of these studies, CD244^−/−^ mice demonstrated increased ability to reject CD48(+) B16 melanoma cells compared with WT. Taken together, these data strongly support an inhibitory role for CD244 in NK cells.

**Figure 3:**
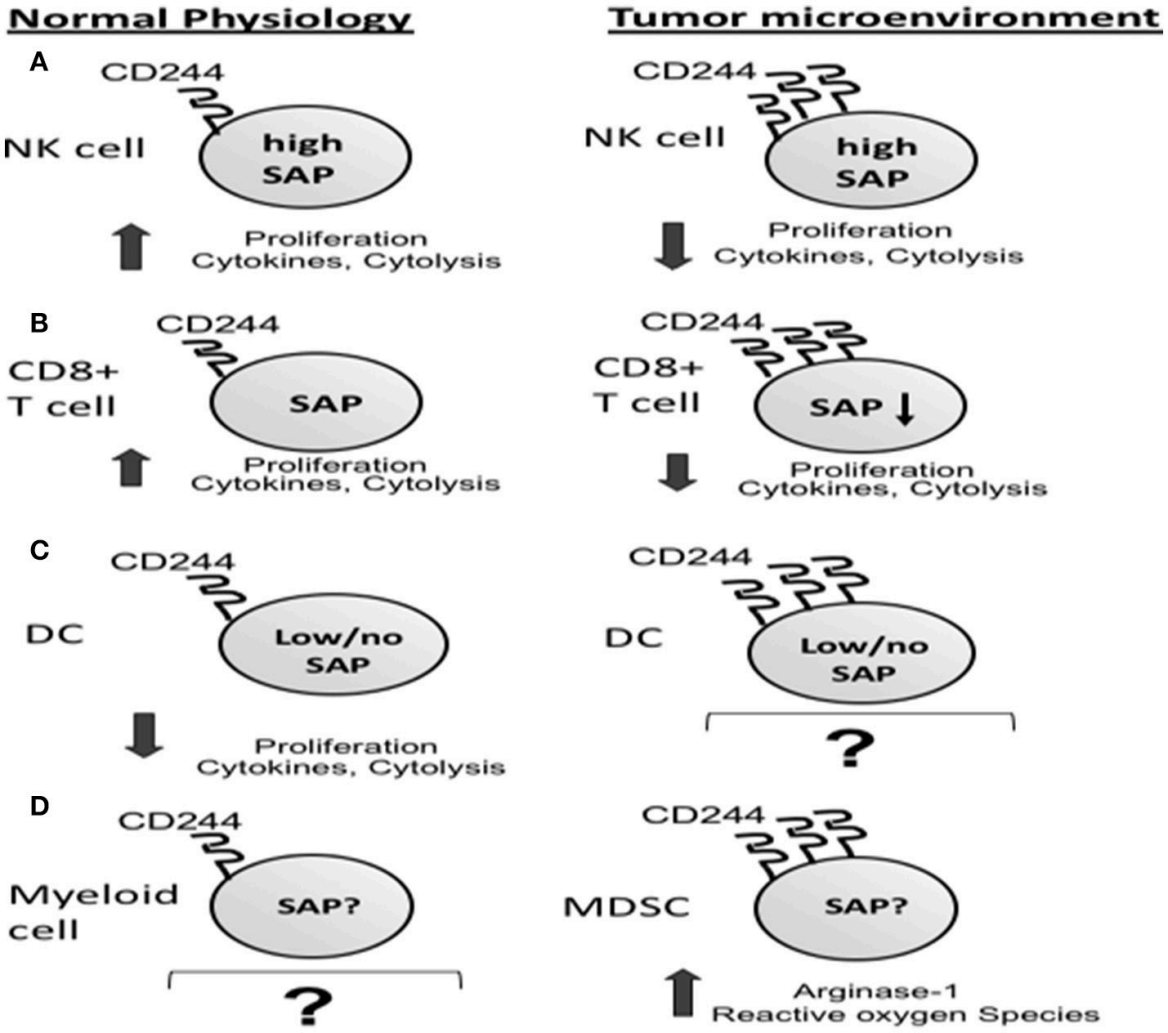
Increased CD244 expression on immune cells in the tumor microenvironment corresponds to increased immunosuppression via effector cell exhaustion **(A,B)** and increased production of immunosuppressors by myeloid derived suppressor cells (MDSCs) **(C)**.

Inhibitory CD244 signaling in NK cells has also been demonstrated in human cancer patients. When CD244+ NK cells were co-cultured with tumor-infiltrating, CD48+CD68+ monocytes/macrophages obtained from patients with hepatocellular carcinoma, they initially demonstrated increased TNFα and IFNγ production, followed by exhaustion with significantly decreased cytokine production and increased apoptosis (9). This exhaustion did not occur in CD244+ NK cells co-cultured with non-tumor liver-infiltrating monocyte/macrophages, which exhibited significantly lower expression of CD48 than those from tumor. Importantly, the exhaustion seen in NK cells co-cultured with tumor-associated CD48^hi^ monocytes/macrophages could be overcome by blocking the CD244-CD48 interaction using anti-CD48 mAb. These observations support a role for CD244 signaling in the development of NK cell exhaustion in the tumor microenvironment (Figure 3A).

## CD244 function in CD8+ T cells

### CD244 signaling and CD8+ T cell exhaustion in chronic infection

CD244 is co-expressed on a subset of antigen-experienced, effector and effector memory CD8+ T cells with other immunoregulatory receptors including PD-1, LAG3, CD160, CTLA-4, and TIM-3 in mice and humans (11, 43–45). In mouse models of chronic viral infection using lymphocytic choriomeningitis virus (LCMV) clone 13, these T-cells exhibit an exhausted phenotype (10, 11). As in NK cells, CD244 receptor concentration affects whether activating or inhibitory signaling occurs. In LCMV-specific CD8+ T cells with low-intermediate CD244 expression, blocking CD244 with anti-CD48 mAb decreases IFNγ production (reflecting activating signaling), while blocking CD244 on exhausted CD8+ T cells with high CD244 expression significantly increases IFNγ production (reflecting inhibitory signaling) (11). Of note, SAP expression decreases in effector CD8+ T cells over time (12), suggesting that, as in NK cells, relative decreases in SAP concentration may lead to inhibitory CD244 signaling in CD8+ T cells. These findings support increased CD244 expression as a mediator of CD8+ T cell exhaustion in the setting of persistent antigen exposure.

In humans, CD244 expression has been shown to be increased on CD8+ T cells in chronic infection (15, 46–51). In these infections, CD244 expression correlates with CD8+CD45RA+CCR7- effector or effector memory subtypes (46, 47). CD244 expression is higher on virus-specific CD8+ T cells in patients with chronic infection than with acute infection and correlates with PD1 expression (15, 47). Human CD8+ T cells with high CD244 expression show evidence of exhaustion that can be reversed with CD244 blockade (15, 47). *Ex vivo*, blockade of CD244 using anti-CD244 mAb on human CD244+CD8+T cells leads to increased virus-specific T cell proliferation, increased expression of CD107a and perforin (markers of degranulation), and IFNy, and increased TNFα and IL-6 in culture supernatants (15, 47, 50, 51). Similarly, the response to CD244 cross-linking *in vitro* seems to depend on the relative expression of CD244, with decreased proliferation in response to CD244 activation in CD244^hi^ CD8+ T cells compared to CD244^lo/int^CD8+ T cells (47). Likewise, the combination of anti-CD3, anti-CD28, and CD244 cross-linking increased IFNγ and degranulation only in CD244^lo^ CD8+ T cells, but not in CD244^hi^ CD8+ T cells. These findings suggest that, as in mice, CD244 in humans is a marker of CD8+ T cell exhaustion in chronic infection, and when expressed at high concentrations, acts as an inhibitory receptor.

### CD244 signaling and CD8+ T cell exhaustion in cancer

In mouse models of cancer, CD244 is expressed on CD8+ T cells with an exhausted phenotype. In syngeneic C57BL/6 mouse models of pancreatic adenocarcinoma and lung carcinoma, frequencies of T-cells exhibiting co-inhibitory receptors CD244, PD-1, and BTLA were increased in tumor-bearing mice compared with naïve controls (52). Consistent with a role in T-cell exhaustion, increased CD244 expression on antigen-specific CD8+ T cells from the spleens of tumor-bearing mice correlated with reduced IL-2 and IFN-γ production (Figure 3B).

In human cancers, CD244 also shows increased expression on exhausted CD8+ T cells. In melanoma, CD244 is increased in tumor-infiltrating lymphocytes compared with peripheral blood, and CD8+ T cells from tumor and peripheral blood show increased CD244 expression compared with CD8+ T cells from healthy controls (43, 44). CD244 is also co-expressed on melanoma-associated CD8+ T cells with other inhibitory receptors, including PD-1, TIM-3 (43, 44), CD160 (43, 45), KLRG1 (44). Because non-hematopoietic cancers do not generally express CD48, T-cell CD244 signaling likely depends on the availability of immune cell-cell interactions within these tumor microenvironments, paralleling the dependence of CD244 signaling in NK cells on local macrophages in hepatocellular carcinoma (9). In multiple myeloma patients, CD8+ T cells demonstrated an exhausted phenotype with decreased CD28 expression, decreased proliferation, and decreased degranulation as measured by mobilization of CD107a (45). These exhausted CD8+ T-cells co-expressed increased CD244, PD-1, CTLA-4, and CD160 compared with non-exhausted CD8+ T cells from healthy controls. Similarly, in AML patients, CD244 expression was increased on peripheral blood T cells compared to healthy controls and correlated with PD-1 expression (53). In another series of AML patients, CD244 expression on T cells was higher in relapse than in new diagnosis, and the degree of CD244 expression in relapsed AML was equivalent to that seen in untreated HIV patients (54). The authors of this second study did not see any correlation between increased CD244 or PD-1 expression and proliferation or cytokine secretion *in vitro*. However, a study by Epling-Burnette et al. demonstrated increased expression of CD244 and decreased expression of activation markers CD28 and CD62L on circulating T cells in myelodysplastic syndrome (MDS) patients, suggesting a link between increased CD244 expression and T cell exhaustion in this segment of the MDS/AML spectrum (55).

## CD244 function in myeloid cells

Dendritic cells are professional antigen-presenting cells that play a crucial role in the induction and maintenance of anti-tumor immunity by cross-presenting tumor antigens and cross-priming tumor-specific T cells. Importantly, the presence of intratumoral DCs is known to correlate positively with prognosis in multiple human cancers (56). However, recent studies have shown that DCs can play an activating or inhibitory role in the tumor microenvironment depending on DC subset, maturation status, and presence of (co-)stimulatory and inhibitory receptors and cytokines (57). Both mouse and human DC populations express CD244. Expression is higher on so-called conventional DC populations compared with plasmacytoid DCs (6). Functionally, CD244^−/−^ DCs from C57BL/6 mice produce significantly higher levels of pro-inflammatory cytokines than WT DCs upon TLR stimulation *in vitro* (6). In addition to priming T cells, DCs contribute to anti-tumor immunity by activation of NK cells. CD244^−/−^ signaling in DCs appears to affect this function: *in vitro*, CD244^−/−^ DCs elicit greater NK cell activation than WT DCs^.^(6) Notably, SAP is not expressed at significant levels in DCs, while EAT-2, SHIP-1, SHP-1, and SHP-2 are all expressed, which may account for the inhibitory role of CD244 signaling in DCs. Further studies are required to elucidate the CD244 signaling pathways in DCs and to determine the influences of the tumor microenvironment on those pathways.

Myeloid-derived suppressor cells (MDSCs) are highly suppressive immune cells found in the tumor microenvironment and in the peripheral blood and spleens of tumor-bearing hosts. Increased numbers of MDSCs have been associated with tumor progression (58, 59), metastases (60, 61), and poor response to current therapies (62–64). Two morphologically distinct subtypes of MDSCs have been identified in both mice and humans: monocytic MDSC (Mo-MDSC: CD11b+ Ly6C^hi^ Ly6G- in mice; CD33+ HLA-DR^lo/−^ CD14+ in humans) and granulocytic MDSC (Gr-MDSC: CD11b+ Ly6C^lo/−^ Ly6G+ in mice; CD33+ HLA-DR^lo/−^ CD15+ in humans) (65). Both subtypes have been shown to produce suppressive mediators including inducible nitric oxide synthase (iNOS), arginase-1, indoleamine 2,3 dioxygenase (IDO), IL-10, and TGFβ1 (66–70). Both MDSC subtypes are known to suppress CD8+ T-cell function (71–73). Recently, the expression of CD244 has been described on MDSCs in tumor-bearing mice, with 30–50% of Gr-MDSC expressing CD244 in four syngeneic tumor models. Distinct differences in Gr-MDSC function were observed between CD244+ and CD244– populations. CD244+ Gr-MDSC significantly suppressed antigen-specific CD8+ T cell response compared to CD244- Gr-MDSC, which did not suppress. Additionally, expression of CD244 on Gr-MDSC correlated with reactive oxygen species (ROS) production and myeloperoxidase (7) (Figure 3C). The correlation between CD244 expression and immunosuppressive capacity in these tumor-associated MDSCs is consistent with the inhibitory role of CD244 signaling in NK cells and CD8+ T cells in the tumor microenvironment.

### Therapeutic considerations and discussion

CD244 is a SLAM family receptor with activating and inhibitory signaling capacities implicated in the functions of NK cells, T cells, DCs and MDSCs in the tumor microenvironment. CD244 appears to predominantly propagate inhibitory signaling in tumor-associated immune cells, but the interplay of factors determining activating versus inhibitory signaling has not been fully elucidated. Increased cell suface density of CD244 and decreased or absent concentrations of functional SAP have both been identified as factors associated with inhibitory signaling (39), while conversely, decreased CD244 density and normal concentrations of functional SAP have been associated with activating signaling (8, 36, 37, 42, 74, 75). In addition, binding to the ITSM domains of the CD244 receptor is a competitive process with SAP preferentially binding over the adaptor molecules associated with inhibitory CD244 signaling (8, 34, 35, 38). These patterns suggest that the ratio of SAP to CD244 is critical to the determination of activating versus inhibitory signaling by this receptor. We theorize that the inhibitory signaling seen in exhausted immune cells with increased CD244 expression occurs because the increased density of CD244 decreases the ratio of SAP to CD244, allowing binding of one of the other adaptor molecules and propagation of an inhibitory signal. Future studies will test this hypothesis in tumor-associated T-cells, DCs, and MDSCs, as differential expression of these molecules is expected to affect the proposed mechanism.

Corresponding to inhibitory signaling in NK cells, CD244 demonstrates an inhibitory function when expressed at higher concentrations on CD8+ T cells that demonstrate an exhausted phenotype in chronic viral infection and cancer (10, 11, 46, 47, 50, 52, 76). Furthermore, in MDSCs from tumor-bearing mice, CD244 expression correlates with suppression of antigen-specific CD8+ T cell function and production of suppressive molecules, suggesting a role for CD244 signaling in the immunosuppressive function of these cells. CD244^−/−^ DCs produce increased levels of pro-inflammatory cytokines and increased activation of NK cells, reflecting inhibitory CD244 signaling in DCs. Taken together, these findings suggest that CD244 signaling on NK cells, CD8+ T cells, DCs, and MDSCs may contribute to immunosuppression in the tumor microenvironment.

The evidence that inhibitory CD244 signaling contributes to immunosuppression in the tumor microenvironment suggests that targeting CD244 could provide a strategy for overcoming resistance to existing checkpoint inhibitors by multiple mechanisms. For example, blocking CD244 signaling on exhausted CD8+ T-cells may ameliorate the exhausted phenotype and contribute to re-activation of memory CD8+ T cells in cancer. Blocking CD244 signaling on DCs may increase pro-inflammatory cytokine release and activation NK cells and CD4+ T cells. Finally, blocking CD244 signaling in MDSCs may decrease the suppressive capacity of these cells, which are known to correspond with more aggressive disease and resistance to current therapies (62–64, 77–79). Importantly, CD244^−/−^ mice appear phenotypically normal with normal maturation of the immune cell repertoire and a normal lifespan compared with other C57BL/6 laboratory mice, suggesting that therapeutic CD244 blockade could be feasible from an adverse effects standpoint. Thus, targeted anti-CD244 therapy could be of benefit as an adjunct to existing checkpoint inhibitors or even conventional chemotherapy strategies with limited myelosuppression.

Besides the effect on immune cells, recent evidence suggests a direct effect of CD244 signaling on CD244-expressing tumor cells. Specifically, CD244 signaling may also play a role in leukemogenesis, adding to its potential as a therapeutic target. A recent study shows that knock-down of CD244 in human leukemia cell lines produces markedly impaired proliferation *in vitro* and *in vivo*, while the repopulation ability of hematopoietic stem cells remains unimpaired following CD244 knockdown (80). Furthermore, in a mouse model of AML, leukemogenesis is dramatically delayed upon CD244 deletion. CD244 may therefore represent a unique therapeutic target if future studies confirm its role as a direct anti-leukemia target and support the hypothesis that it mediates immunosuppressive function in the tumor microenvironment.

## Author contributions

All authors listed have made a substantial, direct and intellectual contribution to the work, and approved it for publication.

### Conflict of interest statement

The authors declare that the research was conducted in the absence of any commercial or financial relationships that could be construed as a potential conflict of interest.
